# Progeroide Syndrome: Alterung, Hautalterung und Mechanismen progeroider Erkrankungen

**DOI:** 10.1007/s00105-023-05212-8

**Published:** 2023-08-31

**Authors:** Kevin Koschitzki, Irina Ivanova, Mark Berneburg

**Affiliations:** Poliklinik und Klinik für Dermatologie, Universitätsklinikum Regensburg, Regensburg, Deutschland

**Keywords:** Pathomechanismen des Alterns, Desoxyribonukleinsäurereparatur, Genodermatosen, Seneszenz, Genetische Mutationen, Pathomechanisms of aging, Deoxyribonucleic acid repair, Genodermatoses, Senescence, Genetic mutations

## Abstract

Progeroide Syndrome (PS) sind durch das vorzeitige Auftreten von altersbedingten Symptomen und Krankheiten gekennzeichnet. PSs sind klinisch heterogen und manifestieren sich unter anderem durch Seh- und Hörverlust, Hautatrophie, Haarausfall, Neurodegeneration, Knochendeformitäten und kardiovaskuläre Defekte. Jüngste Fortschritte in der Molekularpathologie haben zu einem besseren Verständnis der Pathomechanismen dieser Krankheiten geführt. Die verantwortlichen genetischen Mutationen, sind funktionell mit der Erhaltung und Reparatur des Genoms verknüpft, was die ursächliche Rolle der Anhäufung von DNA(Desoxyribonukleinsäure)-Schäden bei der Alterung untermauert. Während einige dieser Gene für Proteine kodieren, die direkt an der DNA-Reparaturmaschinerie beteiligt sind, wie z. B. die Nukleotidexzisionsreparatur (NER), destabilisieren andere das Genom, indem sie die Stabilität der Kernhülle beeinträchtigen, wie z. B. Lamin A beim Hutchinson-Gilford-Progerie-Syndrom (HGPS), oder regulieren die DNA-Schadensreaktion (DDR), wie z. B. das Ataxia-teleangiectasia-mutierte (ATM) Gen. Die Untersuchung der Pathophysiologie progeroider Krankheiten hat zur Entwicklung potenzieller Behandlungen beigetragen.

**Zusatzmaterial online:**

Die Online-Version dieses Beitrags (10.1007/s00105-023-05212-8) enthält eine zusätzliche Tabelle. Beitrag und Zusatzmaterial stehen Ihnen im elektronischen Volltextarchiv auf https://www.springermedizin.de/die-dermatologie zur Verfügung. Sie finden das Zusatzmaterial am Beitragsende unter „Supplementary Material“. Die Tabelle Online-Tabelle 1 ist aus Platzgründen nur elektronisch als Supplementary Information verfügbar.

## Abstract (en)

Progeroid syndromes (PSs) are characterized by the premature onset of age-related pathologies. PSs display a wide range of heterogeneous pathological symptoms that also manifest during natural aging, including vision and hearing loss, atrophy, hair loss, progressive neurodegeneration, and cardiovascular defects. Recent advances in molecular pathology have led to a better understanding of the underlying mechanisms of these diseases. The genetic mutations underlying PSs are functionally linked to genome maintenance and repair, supporting the causative role of DNA damage accumulation in aging. While some of those genes encode proteins with a direct involvement in a DNA repair machinery, such as nucleotide excision repair (NER), others destabilize the genome by compromising the stability of the nuclear envelope, when lamin A is dysfunctional in Hutchinson–Gilford progeria syndrome (HGPS) or regulate the DNA damage response (DDR) such as the ataxia telangiectasia-mutated (ATM) gene. Understanding the molecular pathology of progeroid diseases is crucial in developing potential treatments to manage and prevent the onset of symptoms. This knowledge provides insight into the underlying mechanisms of premature aging and could lead to improved quality of life for individuals affected by progeroid diseases.

Die Alterung ist ein hochkomplexer, multifaktorieller Prozess, den jeder lebendige Organismus durchläuft und der durch einen Rückgang von physiologischen Funktionen und gestörter Homöostase zur Anfälligkeit von Krankheiten, kognitiver Degeneration und schließlich zum Tod führt [1, 2]. Durch die Akkumulation von exogenen und endogenen Schäden kommt es auf zellulärer Ebene zu Telomerverkürzung, Genominstabilität, mitochondrialen Dysfunktionen, Verlust der Proteinhomöostase, epigenetischen Veränderungen, Erschöpfung des Stammzellpools und Seneszenz.

Kommt es durch bestimme Pathomechanismen zu einer Beschleunigung dieser Merkmale und somit einer verkürzten Lebenserwartung, liegt eine progeroide Erkrankung vor [3]. Während sich der physiologische Alterungsprozess in Form von unterschiedlich stark ausgeprägten Alterungssymptomen in fast allen Organsystemen zeigt, kommt es bei progeroiden Syndromen nicht zu einer homogenen Alterung in allen Organsystemen, sondern bestimmte Organsysteme sind durch das Auftreten frühzeitiger Alterungsmerkmale besonders gekennzeichnet. Man spricht deshalb von sog. segmentalen progeroiden Erkrankungen, von denen es über 70 unterschiedliche Erkrankungen gibt. Im Kontrast hierzu stehen nichtsegmentale oder unimodale progeroide Erkrankungen, bei denen es nur in einem Organsystem zur frühzeitigen Alterung kommt [5]. Insgesamt können diese seltenen, monogenen Erkrankungen mit einer heterogenen Klinik wie Hautatrophie, Verlust von subkutanem Fettgewebe, Haarausfall, Osteoporose/Gelenksteifigkeit, kardiovaskuläre Defekte, Neurodegeneration, Hörverlust und ophthalmologische Symptome und Malignität einhergehen. Je nach Entität kann es zudem zu Entwicklungsverzögerungen und Wachstumsstörungen sowie Knochendeformitäten und krankheitstypischen Faziesanomalien kommen [6, 7]. Typische progeroide Symptome sind in Abb. 1 dargestellt.

**Figure 1:**
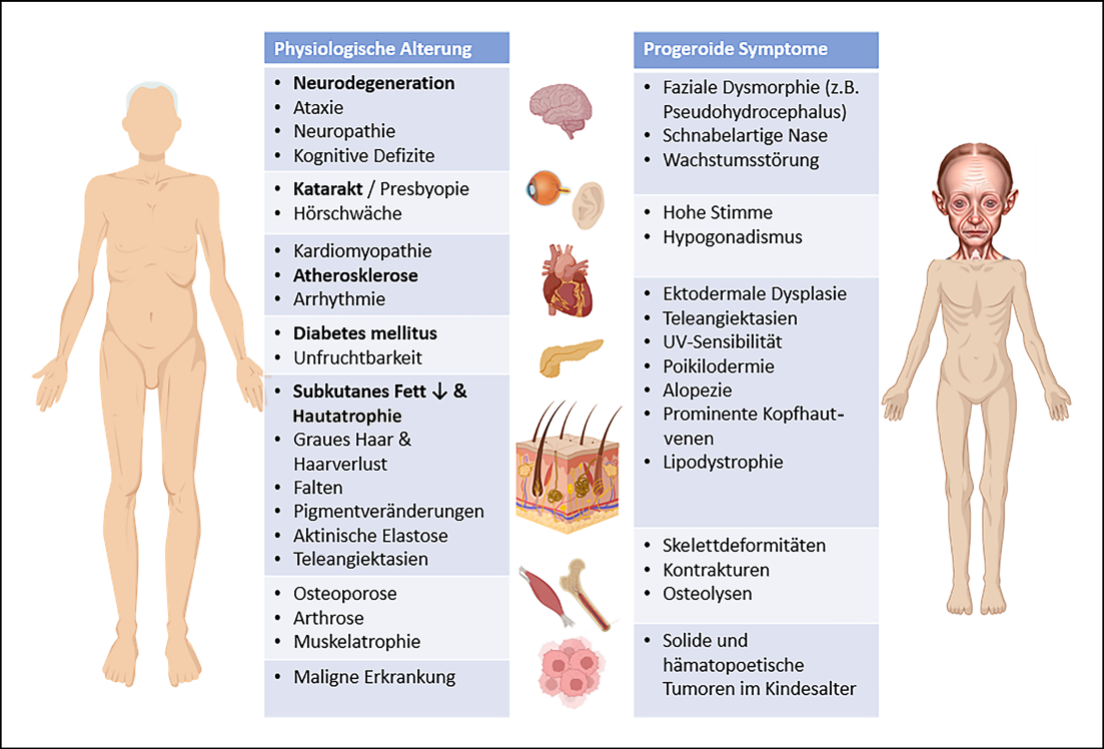

Wie auch bei der natürlichen Alterung spielt die Haut eine besondere Rolle, da sie als Organsystem in direktem Kontakt mit sowohl intrinsischen als auch extrinsischen Stressoren wie UV-Strahlung steht und für jeden objektivierbar ist [8].

## Mechanismen der vorzeitigen Alterung in progeroiden Syndromen

Durch wachsendes Verständnis der Pathophysiologie dieser Erkrankungen gewinnen wir immer mehr Einblicke in die physiologische Alterung und warum es dabei zum Funktionsverlust von Gewebe und Stammzellen kommt. Jedoch sind die zytokinetischen Fehler dieser Erkrankungen komplex, und nicht alle Mechanismen lassen sich mit der Klinik der Erkrankung korrelieren.

In Tab. 1 sind die hier beschriebenen Erkrankungen und deren zugrunde liegenden Mutationen in 3 Pathomechanismen unterteilt, die alle auch bei der physiologischen Alterung eine Rolle spielen, zum Teil aber auch überlappende Charakteristika aufweisen [9].

*Defekte in der Kernlamina* bei sog. Laminopathien führen zu deformierten Zellkernen und Ablagerungen von Präkursorproteinen, wodurch es insbesondere bei epidermalen und mesenchymalen Stammzellen zu Abweichungen in deren Transkription kommt [10].

*Telomere* sind wiederholte DNA(Desoxyribonukleinsäure)-Sequenzen an Chromosomenden, die als schützende Kappen dazu beitragen, Integrität und Stabilität der Chromosomen zu erhalten. Telomere können bei der Zellteilung nicht vollständig durch Telomerasen repliziert werden und verkürzen sich bei jeder Zellteilung, bis das Chromosom instabil wird oder die Replikationsfähigkeit verliert, ein wichtiger Mechanismus der Seneszenz [11]. Bei Telomeropathien bestehen Mutationen im Telomerasekomplex.

**Figure 2:**
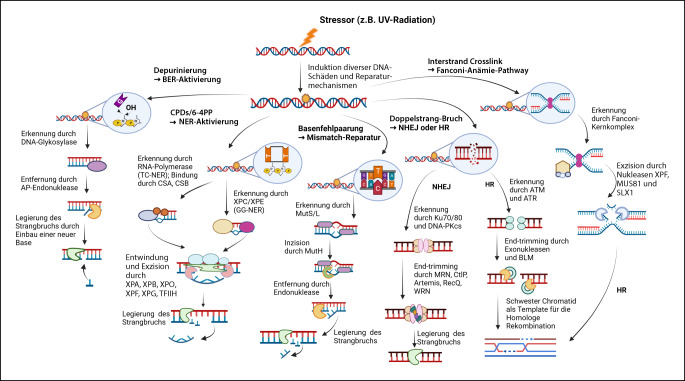

Das* Regenerationspotenzial* spielt eine weitere entscheidende Rolle in der Alterung. Exogene Faktoren wie UV-Strahlung und endogene Faktoren wie oxidative Schäden verursachen täglich 10.000 bis 100.000 Schäden in Form von Einzelstrangbrüchen, Doppelstrangbrüchen und Basenveränderungen an der DNA. Während der Zellteilung sollten diese durch komplexe Reparaturmechanismen, die als „DNA damage response“ (DDR) zusammengefasst werden, erkannt und die Zelle sollte dadurch in den Zellzyklusarrest versetzt werden [12]. Wenn die Akkumulation an Schäden das Maß an Reparaturkapazität der Zelle übertrifft, sollte die Zelle eigentlich durch Apoptose in den geplanten Zelltod übergehen. Tut sie das nicht, kommt es entweder zur im vorherigen Abschnitt beschriebenen Seneszenz oder sie verändert ihre Funktion, und es kommt, falls alle Regulationsprozesse scheitern, zur unkontrollierten Zellproliferation [13]. Die DDR kann in Detektion, Transduktion, Mediation des Signals, Effektor und die Reaktion des Schadens unterteilt werden und interagiert durch Proteinkinasen (ATM [Ataxia-teleangiectasia-mutiert]) mit Tumorsuppressoren (p53) und weiteren Regulatoren [7]. Schadenspezifisch kommt es zur Aktivierung von verschiedenen Reparatursystemen (Abb. 2), wie der direkten Reparatur bei Thymidindimeren und Basenalkylierungen, der Basenexzisionsreparatur (BER) bei Depurinierungen, der Nukleotidexzisionsreparatur (NER) bei UV-Schäden wie Cyclobutan-Pyrimidin-Dimeren (CPDs) und 6‑4-Photoprodukten (6-4PPs), der Mismatch-Reparatur (MMR) bei Replikationsfehlern und Basenfehlpaarung, Fanconi-Anämie-Pathway bei Interstrand Crosslinks (ICLs) und bei Doppelstrangbrüchen zur „homologous repair“ (HR) oder dem „non-homologous end joining“ (NHEJ) [14]. Viele progeroide Erkrankungen werden durch Mutationen relevanter Proteine in dieser Kaskade ausgelöst, was letztendlich zur Akkumulation der Schäden und einer beschleunigten Alterung oder einem erhöhten Krebsrisiko führt [2, 15].

## Defekte in der Kernlamina

### Hutchinson-Gilford-Progerie-Syndrom

Mit der klassischen Progerie wird das Hutchinson-Gilford-Progerie-Syndrom (HGPS) bezeichnet. Bereits im frühen Kindesalter ab 1 bis 2 Jahren beginnen sich die ersten Symptome zu manifestieren [16]. Betroffene Kinder entwickeln neben Wachstumsverzögerungen ein typisch kleines Gesicht mit einer schnabelartigen Nase, Mikrognathie, Alopezie und typischerweise eine hohe Stimme. Zudem bestehen typische progeroide Symptome wie fehlendes subkutanes Fettgewebe und Knochenmineralisierungsdefizite. Im fortschreitendem Alter entwickeln sich koronare und zerebrale Gefäßerkrankungen, die als häufigste Todesursache im frühen Alter von ca. 13 bis 15 Jahren gelten [17].

HGPS wird meistens durch sporadisch autosomal-dominante Mutationen im *LMNA*-Gen verursacht [16], das für Lamin A kodiert, ein wichtiger Bestandteil der Kernlamina. Neben seiner strukturellen Rolle spielt Lamin A eine Rolle in der Mechanotransduktion [18] und interagiert mit Chromatinen sowie deren Regulatoren, Transkriptionsfaktoren und „signaling transduction pathways“, womit das Protein neben der mechanischen Strukturerhaltung ebenfalls zur Organisation des Genoms beiträgt [19]. Mutationen bei HGPS verursachen eine Splicevariante, wobei anstelle von Lamin A aus Prälamin A ein Protein namens Progerin gebildet wird und zu „Blebs“ in der Kernlamina führt. Progerin kommt auch bei der physiologischen Alterung vor und führt zur Instabilität der Kernlamina, Lobulation des Nucleus und Genominstabilität [20]. Bei den Patienten kommt es zusätzlich zur Verkürzung der Telomere [21] und insbesondere bei zellulärem Stress zu den oben erwähnten Mechanismen der Alterung [22]. Die Erkrankung gibt uns viele Einblicke in die Physiologie der Alterung, insbesondere aufgrund der betroffenen mesenchymalen Zelllinien wie Knochen, Muskel und Bindegewebe. Im Kontrast zur physiologischen Alterung und den meisten anderen progeroiden Syndromen liegen hier jedoch kein erhöhtes Krebsrisiko und auch keine Neurodegeneration vor [23].

Bei HGPS wird das Protein Progerin anstatt Lamin A aus dem Precursor Prälamin A gebildet

Eine kausale Therapie existiert nicht. Die einzige aktuell zugelassene Therapie ist weiterhin Lonafarnib, ein Farnesyltransferaseinhibitor (FTI). Farnesylgruppen führen dazu, dass Progerin permanent an der Kernlamina verankert bleibt und seine schädlichen Auswirkungen verursacht. Leider verursachen FTIs selbst zweikernige Zellen, was die Zellproliferation wiederum einschränkt und somit fraglich von Vorteil ist [24]. In einer Studie mit 25 Patienten konnte jedoch ein Drittel der Patienten Gewicht zunehmen, Gelenksteifigkeit reduzieren und die Lebensspanne um 1,6 Jahre verlängern [25]. Während einige Kombinationstherapien mit Lonafarnib sowie weitere Therapieversuche mit „isoprenylcysteine carboxyl methyltransferase“ (ICMT) oder „antisense oligonucleotides“ (ASO) in Tiermodellen Behandlungsoptionen bieten, wird auch Genome Editing wie CRISPR/Cas-Methoden sicherlich eine Rolle in der zukünftigen Behandlung der Erkrankung spielen [26].

### Mandibuloakrale Dysplasie

Die autosomal-rezessiv vererbte mandibuloakrale Dysplasie (MAD) wird anhand der zugrunde liegenden Mutationen in LMNA (MADA) oder ZMPSTE24 in (MADB) unterteilt, welche beide eine Akkumulation an Prälamin A verursachen und damit die Chromatinphysiologie durch unzureichendes Lamin A beinträchtigen [27, 28].

Neben der postnatalen progeroiden Symptomatik sind lokale Osteolysen (Hypoplasie von Mandibula, Klavikula, Finger sowie fliehendes Kinn) und Osteoporose kennzeichnend. Der Hauptunterschied der beiden Untergruppen liegt in der partiellen Lipodystrophie im Bereich der Extremitäten und Fettdepots im Nacken und Gesichtsbereich in Typ A und einer eher generalisierten Lipodystrophie in MADB [29].

### „Néstor–Guillermo progeria syndrome“

Das durch Mutationen in BANF1 („barrier to autointegration factor 1“) ausgelöste „Néstor–Guillermo progeria syndrome“ (NGPS) ist durch eine längere Lebenserwartung und einen langsameren klinischen Verlauf gekennzeichnet [30]. BANF spielt im Auf- und Abbau der Kernlamina während der Mitose eine Rolle, interagiert jedoch auch mit LMNA [31]. Bis heute gibt es nur 3 berichtete Fälle, bei denen jeweils eine normale Entwicklung bis zum 2. Lebensjahr stattfand. Die Klinik ähnelt jedoch stark den zuvor erwähnten MAD-Syndromen. Es kommt zu Wachstumsstörung, progressiver Lipoatrophie und den oben genannten progeroiden Symptomen [5, 32]. Trotz der markanten Skelettveränderungen, kam es zu keinerlei kardiovaskulären Komplikationen, die bei HGPS üblich sind.

### Restriktive Dermopathie

Die auch als „*letale Hyperkeratose-Kontrakturen-Syndrom*“ bezeichnete restriktive Dermatopathie wird meistens durch Mutationen im *ZMPSTE24*-Gen ausgelöst, kann aber auch durch heterozygote De-novo-Mutationen im *LMNA*-Gen hervorgerufen werden [33]. Durch intrauterine Wachstumsretardierung kommt es zu fetaler Akinesie und Polyhydramnion und somit zur Frühgeburt. Betroffene weisen ein typisches Erscheinungsbild mit straffer, dünner Haut mit Hyperkeratosen, Erosionen, Schuppen sowie vorstehende Brustwarzen auf. Es besteht eine charakteristische Fazies mit O‑förmigem Mund, abstehenden Augeninnenwinkeln, kleiner Nase und tief angesetzten Ohren. Knochendeformitäten liegen in Form von überdimensionierten Röhrenknochen, dysplastischen Klavikulae sowie Kontrakturen vor [34]. Durch die fetale Akinesie-Deformation-Sequenz kommt es zur pulmonalen Hypoplasie, weshalb die Kinder innerhalb der ersten Woche den Tod erleiden.

## Störung der Desoxyribonukleinsäurereparatur

### Nukleotidexzisionsreparatur

Die Nukleotidexzisionsreparatur (NER) registriert Einzelstrangbrüche wie sog. „bulky lesions“ oder Photoprodukte wie Cyclobutan-Pyrimidin-Dimere (CPD) oder 6‑4 Photoprodukte (6-4PP) [13]. Man unterscheidet 2 unterschiedliche Initiierungspfade bei der Detektion, die „Global Genome(GG)-NER“ und die „transkriptionsgekoppelte (TC) NER“ die beide in derselben Kaskade enden. Der Hauptunterschied zwischen der langsamen GG- und schnelleren TC-NER besteht darin, dass Erstere Schäden überall im Genom repariert, während TC-NER speziell die Schäden im aktuell transkribierenden Strang repariert [35].

#### Xeroderma pigmentosum

Ein Paradebeispiel für Erkrankungen mit Gendefekten, die an der NER beteiligt sind, stellt die Gruppe der autosomal-rezessiv vererbten Xeroderma-pigmentosum(XP)-Erkrankungen dar, die anhand der Mutation in Komplementationsgruppen XP‑A bis XP‑F unterteilt werden. Insbesondere UV-induzierte CPDs und 6‑4PPs werden nicht wirksam repariert und führen zur Akkumulation von Schäden, Mutationen und Hautkrebs [36].

**Figure 3:**
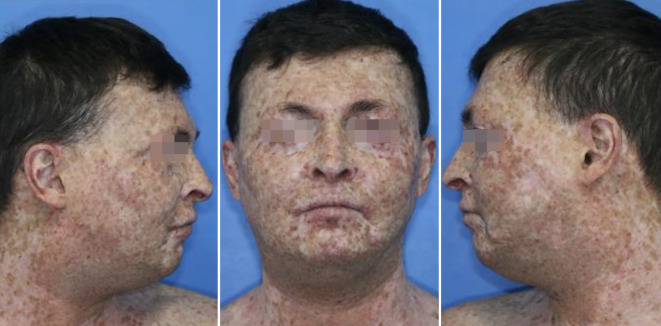

Während Mutationen in XP‑C und XP‑E zu einem Funktionsverlust in der GG-NER führen, bleibt die Funktionsfähigkeit der TC-NER erhalten, was insbesondere zu kutanen Symptomen in Form einer Poikilodermie (Freckling, Hyper- und Hypopigmentierung, Hautatrophie und Teleangiektasien) führt [37]. Insbesondere lichtexponierte Areale wie Gesicht, Nacken, Dekolleté und Unterarme sind hiervon betroffen (Abb. 3). Im Verlauf kommt es zu Basalzellkarzinomen, spinozellulären Karzinomen sowie ophthalmologischen Symptomen. Bei den Betroffenen wurde zudem eine erhöhte Inzidenz an internen Tumoren wie Sarkome oder hepatozellulären Karzinomen beobachtet [38]. XPA, XPB, XPD, XPG und XPF beeinträchtigen die NER erst in späteren Schritten der NER, in denen zusammen mit dem TFIIH-Komplex die DNA-Stränge repariert werden [39]. Klinisch können sich eine frühe Lichtempfindlichkeit, Sonnenbrände und Photophobie sowie zum Teil schwere neurologische Symptome entwickeln, wobei sowohl Entwicklungsverzögerungen als auch Neurodegeneration zu beobachten sind [40]. Der phänotypische Schweregrad variiert zwischen den unterschiedlichen Mutationen und Komplementationsgruppen. Durch eine sog. „unscheduled DNA synthesis“(UDS) lässt sich die NER-Reparaturkapazität in Fibroblasten messen [41]. Insbesondere bei den Untergruppen XP‑A und XP‑D hat sich gezeigt, dass compound-heterozygote Mutationen, bei denen eine Restfunktion der zugehörigen Proteine vorliegt, einen entscheidenden Unterschied in der Entwicklung neurologischer Symptome verursachen [38]. Momentan besteht die einzige Therapiemöglichkeit darin, kompletten Lichtschutz zu betreiben, was neben sozialen, psychologischen und finanziellen Belastungen nicht nur für die Patienten selbst, sondern auch deren Familien eine große Bürde darstellt.

#### Trichothiodystrophie

**Figure 4:**
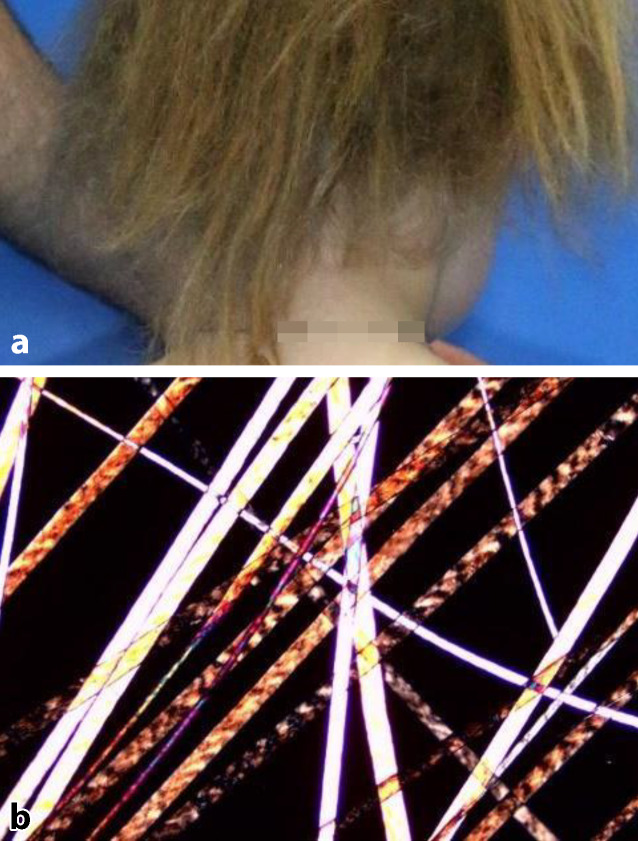

Trichothiodystrophie (TTD) ist wie XP eine sehr heterogene Gruppe von autosomal-rezessiven Erkrankungen. TTD ist durch eine abnormale Cysteinsynthese und somit defiziente Keratine gekennzeichnet. Sie äußert sich durch kurzes, brüchiges Haar (Abb. 4a) (Trichorrhexis nodosa) mit niedrigem Schwefelgehalt und sog. Tiger-Tail-Muster im Polarisationsmikroskop (Abb. 4b) [42]. Die Symptome sind komplex und beinhalten insbesondere neuroektodermale Defekte wie Intelligenzminderung, Nageldystrophie, Katarakte, Fertilitätsstörungen, Kleinwuchs und Anfälligkeit für Infekte [43].

Bislang wurden 10 Gene mit TTD in Verbindung gebracht. Man unterscheidet lichtempfindliche und nichtlichtempfindliche Typen [42].

Etwa die Hälfte der Patienten besitzen Mutationen, die mit einem DNA-Reparaturdefekt in der NER zusammenhängen (XPB/ERCC3, XPD/ERCC2, TTDA/GTF2H5) und für Subgruppen des Transkriptionsfaktors IIH (TFIIH) kodieren, was eine Rolle in der Transkriptionsinitiierung spielt. Diese Subgruppen werden momentan noch als „lichtempfindlich“ klassifiziert, wobei der klinische Phänotyp häufig nicht zwangsläufig mit einer UV-Empfindlichkeit korreliert [44, 45]. Insbesondere bei diesen Patienten sollte auf Hauttumoren geachtet werden und ggf. eine Messung der NER (UDS) durchgeführt werden. Wie auch bei XPD gibt es hier compound-heterozygote XPD/ERCC2-Mutationen mit Restproteinfunktion und einer erstaunlich milden Hautsymptomatik. Die andere Hälfte wird als nicht lichtempfindlich klassifiziert, und die NER-Funktion der Patienten bleibt unbetroffen [42]. Bei den 7 identifizierten Genen dieser Formen liegen Mutationen im M‑Phasen-spezifische PLK1-interagierenden Protein (MPLKIP/TTDN1), der allgemeinen Transkriptionsfaktor-IIE-Untereinheit‑2 (GTF2E2/TFIIEβ), dem X‑gebundenen Ringfingerprotein 113A (RNF113A) und Cysteinyl-tRNA-Synthetase 1 (CARS1), Threonyl-tRNA-Synthetase 1 (TARS1), Alanyl-tRNA-Synthetase (AARS1) und Methionyl-tRNA-Synthetase (MARS1) vor [42, 44, 46]. Da die Klinik anhand der Mutationen sehr stark variiert, ist eine aktuellere Klassifizierung der Erkrankung anhand des Genotyps dringend notwendig.

#### Cockayne-Syndrom

Das Cockayne-Syndrom (CS) wird in erster Linie durch Mutationen in den Genen *CSA* (ERCC8) oder *CSB* (ERCC6) verursacht. CSB (ERCC6-Gen Lokus 10q11) wird in der Literatur auch als DeSanctis-Cacchione-Syndrom bezeichnet. Beide Gene kodieren Proteine, die in der TC-NER involviert sind [47]. Klinisch können sich die Symptome selbst bei homozygoten Varianten interfamiliär variabel zeigen [48]. Zusammenfassend geht die Erkrankung jedoch im Vergleich zu XP insbesondere mit einer fortschreitenden Neurodegeneration ähnlich wie bei TTD einher. Mikrozephalie und Wachstumsstörung sind kardinale Symptome [49]. Die Patienten können neben Ataxie, Tremor und Dystonie zudem Wachstumsstörungen, Intelligenzminderung, Mikrozephalie, Lichtempfindlichkeit, Hörverlust und Netzhautdegeneration aufweisen. Die allgemeine Überlebenserwartung liegt bei ca. 8 bis 9 Jahren.

Neurodegenration, Mikrozephalie und Wachstumsstörung sind kardinale Symptome des Cockayne-Syndroms

Liang et al. konnten neuerdings eine Verbindung von CSB-Funktionsverlust mit der Hyperaktivierung von Necdin, einem Melanoma-assoziierten Antigen aufzeigen [50]. Necdin ist sowohl in der Neurogenese als auch für das Überleben von postmitotischen Neuronen relevant, spielt aber keine Rolle in der UV-induzierten TC-NER. Zudem scheint beim Cockayne-Syndrom die ribosomale RNA-Homöostase eingeschränkt zu sein [51]. Beides sind Faktoren, die zur schweren neurologischen Symptomatik beitragen.

**Figure 5:**
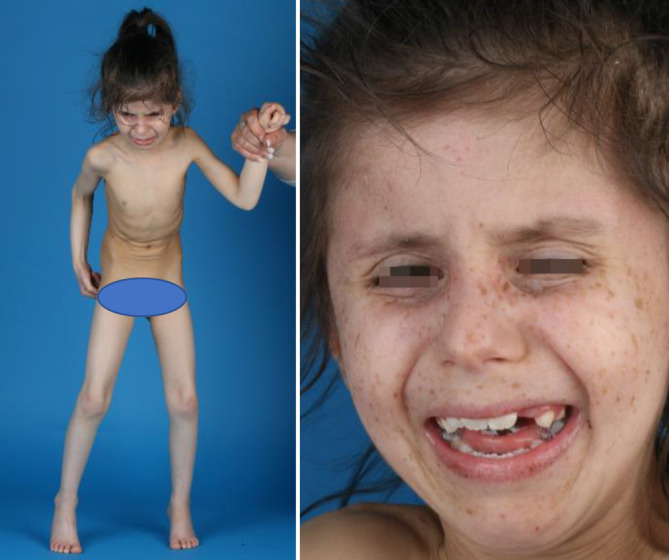

Neben Mutationen in CSA und CSB existieren noch seltenere Kombinationen bzw. Überlappungssyndrome aus Mutationen mit Xeroderma pigmentosum (XP/CS), wie z. B. in ERCC5, welches das Protein XP‑G kodiert, „TFIH complex“ (Trichothiodystrophie/CS) und ERCC1 (XPF/CS), das auch als „Cerebrooculo-facio-skeletal“-Syndrom (COFS) bekannt ist [52]. Das Krebsrisiko bei XP/CS-Syndromen (XPB, XPD, XPG) ist im Vergleich zu CS deutlich erhöht ([40, 53]; Abb. 5).

### Fanconi-Anämie-Pathway

#### Fanconi-Anämie

Die Fanconi-Anämie (FA) ist eine Gruppe von genetisch und klinisch heterogenen Erkrankungen aus 8 verschiedenen Komplementationsgruppen. Es konnten Mutationen in mindestens 23 verschiedenen Genen identifiziert werden, die an dem DNA-Reparaturweg „Fanconi-Anämie-Pathway“, beteiligt sind [54].

Der hochkomplexe Mechanismus aus diesen 23 Proteinen löst kovalente Bindungen, die „Interstrand Crosslinks“ (ICLs) auf, die durch exogene Karzinogene oder auch DNA-Crosslinker, wie z. B. Cisplatin, gebildet werden und besonders toxisch auf Zellen wirken, indem sie chromosomale Instabilität verursachen [55]. Der Pathway wird durch die Bildung eines Reparatur-Kernkomplexes initiiert, der für die Erkennung des Schadens verantwortlich ist. Anschließend werden Nukleasen rekrutiert, die die Stränge trennen. Es erfolgt die Transläsionssynthese, um anschließend mittels homologer Rekombination den Strang zu vervollständigen. Der FA-Weg koordiniert somit auch die NER, HR und NHEJ [56]. Mutationen in FA-Genen stören die Funktion des Signalwegs, was zu einer Anhäufung von Schäden und somit zur chromosomalen und genomischen Instabilität führt [54].

Klinisch kennzeichnend ist ein Knochenmarkversagen in Form einer Panmyelopathie innerhalb der ersten Lebensjahre. Zudem bestehen Entwicklungsstörungen und typische Hyperpigmentierung, Hypopigmentierungen und Café-au-lait-Flecken. Es besteht ein erhöhtes Risiko für akute Leukämien (AML [akute myeloische Leukämie], MDS [myelodysplastisches Syndrom]), Lebertumoren und spinozelluläre Karzinome, was zu einer reduzierten Lebenserwartung führt. Zudem kann es zu Wirbelkörperanomalien, Analatresie, kardialen Defekten, tracheoösophagealen Fisteln, renalen Missbildungen sowie Missbildungen der Extremitäten und des Schädels kommen [54]. Durch Stammzelltransplantation, die als Standardtherapie der FA eingesetzt wird, konnte insgesamt das 3‑Jahres-Überleben um 80 % bei HLA-kompatiblen Geschwistern angehoben werden. Dadurch steigt jedoch auch das Risiko bezüglich SCCs (Plattenepithelkarzinome) und hepatozellulärer Karzinome. Patienten sollten auf keinen Fall Alkohol trinken oder rauchen. In Zukunft könnten Gentherapie und Gen-Editing, wie z. B. CRISPR-Cas9, eine Rolle in der Therapie spielen [54].

### „Homologous repair“

#### Bloom-Syndrom

Das autosomal-rezessiv vererbte Bloom-Syndrom (BS) entsteht durch homozygote Mutationen im *BLM*-Gen, das für ein RecQ-Helikase-Enzym kodiert. Die RecQ-Helikase-Familie besteht aus 5 Helikasen: BLM (Bloom-Syndrom), WRN (Werner-Syndrom), RecQ1, RecQ4 (Rothmund-Thompson-Syndrom) und RecQ5 [57]. Diese Helikasen sind für das Aufbrechen der Wasserstoffbrückenbindungen zwischen den Basenpaaren und somit für die Konversion der Doppelhelix in Einzelstränge verantwortlich [58].

Das BLM-Protein ist dabei an der Stranginversion zwischen DNA-Replikation/Reparatur bzw. Replikationsreinitiierung beteiligt. Die Mutationen führen zu einer Genominstabilität, durch die eine erhöhte Rate von Austausch zwischen 2 Schwesterchromatiden in Fibroblasten und Lymphozyten beobachtet wurde [57]. Klinisch äußert sich das Syndrom insbesondere durch die hohe Anzahl an somatischen Mutationen und Malignität mit einer Prädilektion für Leukämien, Lymphome und solide Tumoren der Haut und des Gastrointestinaltrakts [59]. Neben prä- und postnatalen Wachstumsstörungen kommt es zu einem typisch schmalen, länglichen Gesicht mit Dolichozephalie. Die UV-empfindliche Haut wirkt teleangiektatisch-erythematös in lichtexponierten Arealen. Zudem bestehen Immundefizienz und Infektanfälligkeit [60].

#### Rothmund-Thomson-Syndrom

Das *RecQl‑4*-Gen-assoziierte Rothmund-Thomson-Syndrom (RTS) ist eine autosomal-rezessiv vererbte Störung die durch ein marmorartiges, teleangiektatisches Gesichtserythem gekennzeichnet ist, sich auf Extremitäten, palmar und plantar ausbreitet und im Verlauf ein poikilodermatisches Erscheinungsbild erzeugt [61]. Es wird zwischen RTS‑I und RTS-II unterschieden. Für RTS‑I sind neben Poikilodermie und Lichtempfindlichkeit insbesondere ektodermale Dysplasie (Radiusaplasie) und juvenile Katarakte charakteristisch. RTS-II geht neben Poikilodermie mit Knochendefekten einher und hat ein erhöhtes Malignitätsrisiko, insbesondere hämatoonkologischer Natur [62]. Gesichtsdeformitäten sind zu Geburt oft nicht vorhanden oder unspezifisch. Wachstumsstörungen, Kleinwuchs, Haarlosigkeit geben den Hinweis auf ein progeroides Syndrom [63].

### „Nonhomologous end-joining“

#### Werner-Syndrom

„Nonhomologous end-joining“ (NHEJ) ist ein Mechanismus der DNA-Reparatur, der die direkte Ligation gebrochener DNA-Enden beinhaltet, ohne dass eine homologe Vorlage benötigt wird [64].

Das autosomal-rezessiv vererbte Werner-Syndrom wird unterteilt in ein juveniles und ein adultes Progeriesyndrom. Es beginnt mit Symptomen in der Adoleszenz [65]. Erst ab der Pubertät oder einer Pubertas tarda fangen die Patienten an, „schnell zu altern“. Es kommt zu Katarakten, grauen, dünnen Haaren und Atrophie des subkutanen Fettgewebes sowie Sklerodermie. Alterserkrankungen wie Typ-2-Diabetes mellitus, Hypogonadismus, Osteoporose und Atherosklerose sowie seltenere tumoröse Erkrankungen wie beispielsweise Sarkome äußern sich bereits früher [66]. Charakteristisch sind die Entwicklung einer vogelartigen Gesichtsfazies und Ulzerationen im Bereich der Fußknöchel [67]. Das *WRN*-Gen auf Chromosom 8 kodiert ebenfalls für eine RecQ-Typ-Helikase und ist somit ebenfalls an der Doppelstrangbruchreparatur beteiligt. Allerdings ist WRN das einzige Protein mit einer Exonukleasefunktion und übernimmt neben DNA-Reparatur, Rekombination und Replikation auch eine regulatorische Rolle in der Telomerintegrität [66].

#### Ataxia teleangiectasia

Autosomal-rezessiv vererbte Mutationen im *ATM*-Gen verursachen Einschränkungen in der ATM-Kinase-Aktivität, eine Serinproteinkinase, die insbesondere als Transducer von Doppelstrangbrüchen und Aktivator von Tumorsuppressoren wie beispielsweise p53 und weiteren Zellzykluskontrollpunkten dient. Somit ist das Protein zentral bei der Instandhaltung der Genomstabilität durch die nichthomologe Endverbindung (NHEJ) beteiligt [68]. Etwa 1300 Fälle von Ataxia teleangiectasia (AT) oder „Louis-Bar-Syndrom“ sind in der Literatur beschrieben. Man unterscheidet die klassische (und schwerste) Form der AT, die insbesondere durch eine Ataxie und Motordysfunktion aufgrund zerebellärer Degeneration durch den Verlust von Purkinje-Zellen gekennzeichnet ist, von AT-Varianten, bei denen noch ein Rest an Kinaseaktivität vorhanden ist und die eine heterogene klinische Präsentation aufweisen [69]. Weitere neurologische Symptome bestehen aus Gelenkschmerzen, Schluckstörungen, Dystonie, Tremor und peripheren Neuropathien. Diese können sich im Verlauf progressiv entwickeln [70]. Zudem kommt es zu namensgebenden fazialen und konjunktivalen Teleangiektasien. Außerdem können eine Immundefizienz mit Thymusdegeneration und endokrinologischen Erkrankungen sowie Ovarialinsuffizienzen bestehen. Es besteht eine hohe Empfindlichkeit gegenüber Schäden durch ionisierende Strahlung. Letzteres spielt klinisch eine entscheidende Rolle. Die Lebenserwartung ist insbesondere durch hämatologische Malignitäten wie Leukämien und Lymphome mit T‑Zell-Prädominanz reduziert [71]. Die Wahrscheinlichkeit für solide gastrointestinale, Haut- und Brusttumoren steigt insbesondere bei älteren Patienten [72].

## Telomeropathien

Telomere schützen die Endkappen der Chromosomen im Sinne eines Shelterin-Komplexes, der das Erkennen von DNA-Doppelstrangbrüchen (DSBs) verhindert. Sollten die Telomere dennoch als DSBs erkannt werden, kommt es zu einem Stopp im Zellzyklus. Telomere haben einen eigenen Replikationsapparat, der aus TERT und TERC besteht. Kommt es zur Verkürzung von Telomeren, trägt dies zu Seneszenz und Alterung bei [73]. Mehr als 14 Genmutationen wurden mit einer Dyskeratosis congenita in Verbindung gebracht (DKC1, TERC, TERT, NOP10, NHP2, TINF2, C16orf57/USB1, TCAB1, CTC1 und RTEL1) [74]. Die häufigste davon, die klassische X‑chromosomal-rezessive Form zeigt Mutationen in DKC1, das für Dyskerin kodiert und am Aufbau des Telomerasekomplexes beteiligt ist. Sie kommt häufiger bei Männern vor. Mutationen in TERT und TERC kodieren für Telomerase-Reverse-Transkriptase bzw. RNA(Ribonukleinsäure)-Komponenten. NOP10 und NHP2 sind für kleine nukleoläre Ribonukleoproteine (snoRNP) verantwortlich, und WRAP53 sowie C16orf57 sind an der Ribosomenbiogenese beteiligt. NOP10, NHP2, WRAP53 und C16orf57 sind an der Erhaltung der Telomere beteiligt [75].

Klinisch wird eine klassische Symptomtrias aus retikulärer Hyperpigmentierung im Nackenbereich, Nageldystrophie und Leukoplakie beschrieben. Jedoch resultiert der Schweregrad des heterogenen Erkrankungsbildes aus hämatoonkologischen und soliden Tumoren, Knochenmarkversagen, Lungen- und Lebererkrankungen sowie einer progredienten Immunschwäche [62]. Der Schweregrad des Phänotyps ist direkt korrelierbar mit dem Schweregrad der Telomerverkürzung. So kann sich die Erkrankung zum Teil erst in der späten Jugend oder im jungen Erwachsenenalter äußern oder wie bei Høyeraal-Hreidarsson (HH), das als Maximalvariante der Dyskeratosis congenita (DKC) gilt, bereits intrauterin beginnen [76].

## Störungen von zellulärer Signalübertragung und Stoffwechsel

Obwohl einige der oben genannten Syndrome ebenfalls Veränderungen von extrazellulärer Matrix, Golgi-Apparat, Mitochondrien und Ribosomen verursachen, gibt es Erkrankungen, die sich insbesondere durch einen Pathomechanismus in Stoffwechselorganellen äußern. Ein Beispiel ist das Marfan-Progeroid-Lipodystrophie-Syndrom (MPLS), das durch Mutationen in FBN1 verursacht wird. Hier sorgt die Mutation in Fibrillin 1 für eine kongenitale, schwere Lipodystrophie, progeroide Fazies und hypermobile Gelenke, Myopie sowie weitere Symptome des Marfan-Syndroms [77]. Beim autosomal-rezessiven Berardinelli-Seip-Syndrom (BSS) – oder kongenitale generalisierte Lipodystrophie – bestehen Dysregulationen des Lipid- und Kohlenhydratmetabolismus. Klinisch besteht fast eine komplette Atrophie des subkutanen Fetts, und Erkrankte entwickeln schnell metabolische Erkrankungen wie Diabetes mellitus, Pankreatitis und Hepatosteatosis [78]. Auch bei Cutis-laxa-Erkrankungen, einer seltenen Gruppe von Bindegewebserkrankungen, kommt es aufgrund von komplexen Defekten in Synthese, Aufbau und Abbau von Elastin zu schlaffer, faltiger Haut ohne Elastizität. Die autosomal-rezessiven Varianten (ARCL) sind durch eine starke Beteiligung der extrazellulären Matrix gekennzeichnet und können progeroide Züge zeigen. Es kommt zu intrauterinen Wachstumsstörungen, Kleinwuchs und typischerweise einem dreieckigen Gesicht mit tiefliegenden Augen und kleinem Mund mit spitzem Kinn sowie hypoplastischem Oberkiefer [80]. ARCL‑1 (FBLN4, FBLN5, LTBP4) wird durch Mutationen in ECM-Proteinen verursacht und verursacht schwere kardiopulmonale Komplikationen. ARCL‑2 (ATP6V0A2) kodiert für eine lysosomale ATPase, und auch bei ARCL‑3 (PYCR1 & ALDH18A1) spielen die Proteine insbesondere im Stoffwechsel von Golgi-Apparat, Mitochondrien und Endosomen eine Rolle. Insbesondere ARCL‑3 ist durch dünne atrophe Haut im Bereich der Kopfhaut gekennzeichnet.

## Fazit für die Praxis

- Progeroide Syndrome sind durch das vorzeitige Auftreten von altersbedingten Symptomen und Krankheiten gekennzeichnet.
- Progeroide Erkrankungen äußern sich klinisch sehr heterogen und manifestieren sich unter anderem durch Kleinwuchs, Seh- und Hörverlust, Hautatrophie, Haarausfall, Neurodegeneration, Knochendeformitäten und kardiovaskuläre Defekte sowie metabolische Erkrankungen und Krebs.
- Durch wachsendes Verständnis der Pathophysiologie dieser Erkrankungen gewinnen wir immer mehr Einblicke in die physiologische Alterung und warum es dabei zum Funktionsverlust von Gewebe und Stammzellen kommt. Diese Erkenntnise haben auch zur Entwicklung potenzieller Behandlungen beigetragen.

## Supplementary Information




